# Evaluation of the Molecular Mechanisms of Sepsis Using Proteomics

**DOI:** 10.3389/fimmu.2021.733537

**Published:** 2021-10-21

**Authors:** He Miao, Song Chen, Renyu Ding

**Affiliations:** ^1^ Department of Intensive Care Unit, The First Hospital of China Medical University, Shenyang, China; ^2^ Department of Trauma Intensive Care Unit, The First Affiliated Hospital of Hainan Medical University, Haikou, China; ^3^ Key Laboratory of Emergency and Trauma of Ministry of Education, Hainan Medical University, Haikou, China

**Keywords:** sepsis, proteomics, cecal ligation and puncture, lipopolysaccharide, post-translational modifications

## Abstract

Sepsis is a complex syndrome promoted by pathogenic and host factors; it is characterized by dysregulated host responses and multiple organ dysfunction, which can lead to death. However, its underlying molecular mechanisms remain unknown. Proteomics, as a biotechnology research area in the post-genomic era, paves the way for large-scale protein characterization. With the rapid development of proteomics technology, various approaches can be used to monitor proteome changes and identify differentially expressed proteins in sepsis, which may help to understand the pathophysiological process of sepsis. Although previous reports have summarized proteomics-related data on the diagnosis of sepsis and sepsis-related biomarkers, the present review aims to comprehensively summarize the available literature concerning “sepsis”, “proteomics”, “cecal ligation and puncture”, “lipopolysaccharide”, and “post-translational modifications” in relation to proteomics research to provide novel insights into the molecular mechanisms of sepsis.

## Introduction

Sepsis is a life-threatening multiple organ dysfunction resulting from a dysregulated host response to infection, including acute respiratory distress syndrome (ARDS), acute kidney injury (AKI), or disseminated intravascular coagulation (DIC) (1, 2). Sepsis is characterized by high morbidity and mortality. The Global Burden of Disease study identified 48.9 million cases of sepsis worldwide in 2017 (3). However, it is difficult to accurately estimate the incidence of sepsis because of the underreporting of cases in medically underdeveloped countries. Furthermore, because of an increasingly aging society in many countries, the occurrence of sepsis is likely to be on the rise (4). Although the guidelines for the diagnosis and treatment of sepsis have made great progress in the past decade and the prognosis has been improved, the mortality rate remains high. More than 25%–30% of patients with sepsis die of the disease, and the in-hospital mortality rate of septic shock is close to 40%–60% (4, 5). Therefore, a deep understanding of the biological mechanism, early and accurate diagnosis, and effective treatment of sepsis is essential.

In the last decade, various omics techniques have been used for the study of sepsis, including genomics (6, 7), transcriptomics (8–10), proteomics (7), and metabolomics (7, 11). Since the completion of the Human Genome Project and accumulation of extensive genomic data, proteomics has become an integral component of the post-genomic era (12). The essence of proteomics is to study the characteristics of proteins on a large scale, including protein identification, post-translational modifications (PTMs; glycosylation, phosphorylation, etc.), and protein function determination (13). There are many research methods for proteomics, such as chromatography-based techniques (traditional techniques) (14) and protein chips (advanced technologies) (14, 15). However, each technology has its advantages and limitations. The following are the two most common approaches for proteomics in sepsis. The first is to search for biomarkers, which helps in the early diagnosis of sepsis and organ function damage caused by sepsis through proteomics (16). The second is to explore the molecular mechanism of sepsis and sepsis-related organ function damage by comparing the differences or dynamic changes in protein expression between sepsis and control patients to identify therapeutic targets, thereby achieving precision medicine. Herein, we reviewed proteomics studies published in the past two decades using the keywords “sepsis,” “proteomics,” “cecal ligation and puncture” (CLP), “lipopolysaccharide” (LPS), and “post-translational modifications.” We summarize the application of proteomics in elucidating the molecular mechanism and potential therapeutic targets of sepsis and the research progress of protein PTMs in sepsis. In addition, we discuss the potential problems and development prospects of proteomics.

## Pathophysiological Mechanisms of Sepsis

The detailed complex pathophysiological mechanisms underlying sepsis remain elusive; however, inflammatory and immune responses appear to play key roles in this process. Pathogens activate immune cells by interacting with pattern-recognition receptors (e.g., Toll-like receptors [TLRs]) and regulate the expression of proinflammatory factors *via* signaling pathways such as the TLR/MyD88/NF-κB and TLR/Trif/IRF3 pathways. These receptors recognize the structures of pathogenic microorganisms, known as pathogen-associated molecular patterns (PAMPs). The same receptors also recognize endogenous molecules released from injured cells, known as damage-associated molecular patterns (DAMPs), such as histones, extracellular DNA, and heat shock proteins (HSPs) (17–19). In general, proinflammatory reactions are directed at eliminating invading pathogens, whereas anti-inflammatory responses help to limit the degree of local and systemic tissue injury (20). Therefore, an imbalance between proinflammatory reactions and anti-inflammatory responses results in a series of uncontrolled host responses, including systemic inflammatory response syndrome, coagulation abnormalities, immunosuppression, neuroendocrine disorders, and metabolic disorders (17, 21–23).

Coagulation abnormalities are common in sepsis (24). Proinflammatory mediators induce the expression of tissue factors and promote the cascade of coagulation pathways. Activity of the anticoagulant system (e.g., protein C and antithrombin) is reduced, and the increase in anti-fibrinolytic plasminogen activator inhibitor-1 levels inhibits the activation of the fibrinolytic system (20). These changes cause the formation of microthrombi, resulting in sepsis-induced coagulopathy (SIC) and even DIC (18). Neutrophil extracellular traps (NETs) (19), endothelial cell injury (25), and platelet activation (26) are all involved in the pathophysiological process of SIC.

The apoptosis of lymphocytes and antigen-presenting cells is the main pathogenic event that contributes to immunosuppression in sepsis (27). Immune checkpoints, represented by programmed death (PD)-ligand 1/PD-1, play an important role in the regulation of immune cell apoptosis (19, 28). Myeloid-derived suppressor cells, which are produced in high quantities during sepsis, secrete anti-inflammatory cytokines, such as interleukin (IL)-10 and transforming growth factor-β, which aggravate immunosuppression (18). Impaired phagocytic function of macrophages and neutrophils and decreased expression of HLA-DR on the surface of monocytes also contribute to immunosuppression in sepsis (18).

In sepsis, the sympathetic adrenomedullary system is excited and produces large amounts of catecholamine neurotransmitters, which further increase the expression and release of proinflammatory factors, thereby aggravating endothelial cell injury (17). The cholinergic anti-inflammatory pathway associated with the vagus nerve plays a role in antagonizing the inflammatory response (20). The hypothalamus is the regulatory center of the neuroendocrine and autonomic nervous systems. The function of the hypothalamic–pituitary–adrenal axis is impaired during sepsis, resulting in relative adrenal insufficiency (29). Similarly, sepsis disturbs thyroid hormone synthesis and secretion, which causes low T3 syndrome (27).

Changes in hormone levels during sepsis can also cause metabolic disorders. The classical hormone-induced metabolic disorder is stress-induced hyperglycemia, which is associated with insulin resistance and increased glucocorticoid and glucagon levels (30). Other sepsis-related metabolic disorders include fatty acid/amino acid metabolism disorders, anaerobic metabolism, oxidative stress, and abnormalities in energy metabolism. Mitochondria are the power plants of cells that produce adenosine triphosphate (ATP) *via* the tricarboxylic acid (TCA) cycle and maintain cellular function (31). Mitochondrial dysfunction leads to the generation of large amounts of reactive oxygen species (ROS) and can induce cell death (e.g., mitoptosis), which plays an important role in the mechanism underlying the pathogenesis of sepsis (31). Notably, glucose metabolism pathways, including glycolysis, the TCA cycle, and oxidative phosphorylation (OXPHOS), are closely related to the function of immune cells (i.e., immune metabolism) (32). For example, in sepsis, macrophages are divided into two subtypes: M1 and M2. M1 macrophages generally exert proinflammatory effects by secreting proinflammatory factors (33, 34) with a cellular metabolism mode dominated by glycolysis, relatively low OXPHOS activity, and high inducible nitric oxide synthase (iNOS) activity (35). By contrast, M2 macrophages dominate during the resolution of inflammation by secreting anti-inflammatory factors and participating in processes linked to immunosuppression and tissue repair (34). M2 macrophages rely on enhanced OXPHOS and the intact TCA cycle to support their metabolic program (36). Thus, these uncontrolled host responses are interrelated and collectively contribute to the pathophysiological process of sepsis.

**Figure 1:**
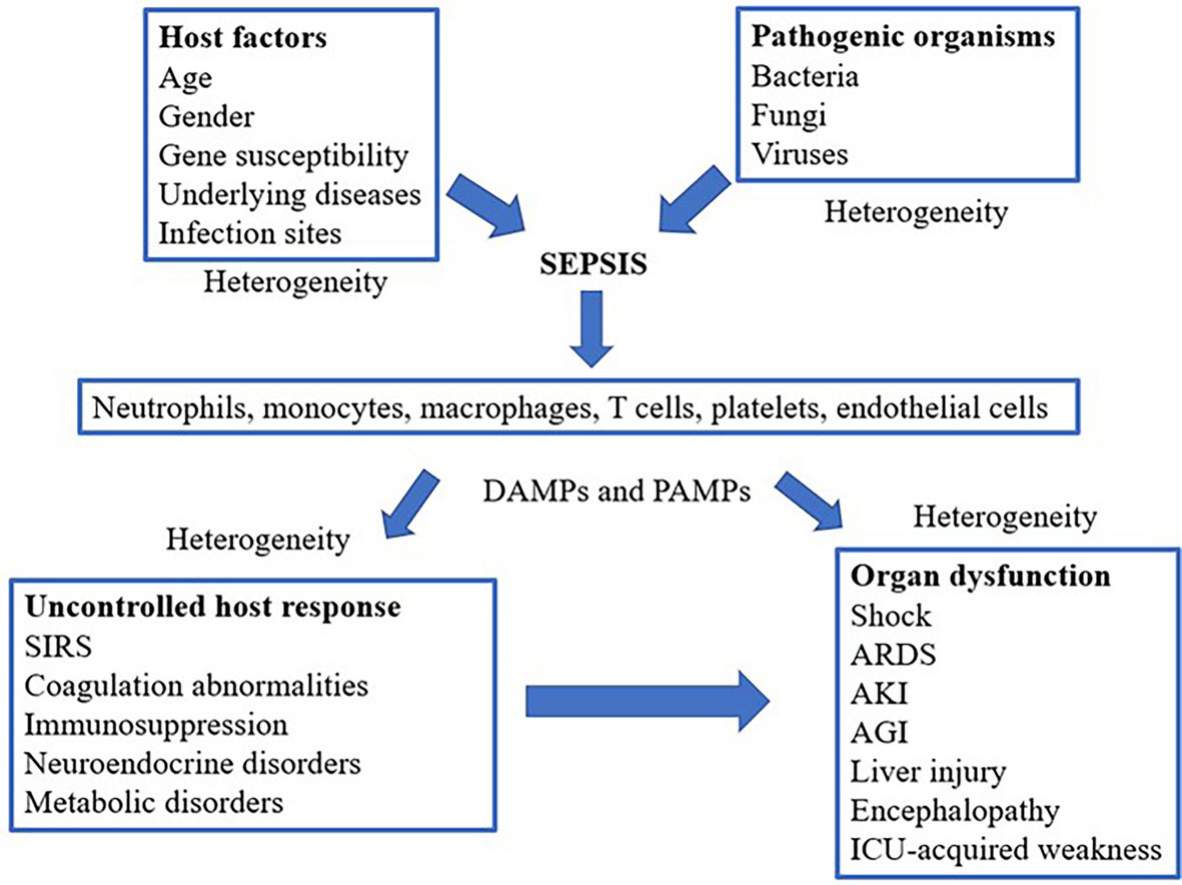
Schematic mechanism of sepsis, including cell types involved and the pathophysiological processes leading to organ dysfunction. SIRS, systemic inflammatory response syndrome; ARDS, acute respiratory distress syndrome; AKI, acute kidney injury; AGI, acute gastrointestinal injury; DAMPs, damage-associated molecular patterns; PAMPs, pathogen-associated molecular patterns; ICU, Intensive Care Unit.

Such uncontrolled host responses and the accompanying production of inflammatory mediators can damage endothelial cells present in diverse tissues and organs, causing organ dysfunction or even failure (37, 38) (**Figure 1**). The main processes contributing to organ dysfunction in sepsis are as follows (20, 22, 39). First, endothelial cells play an essential role in maintaining organ homeostasis, including vasoregulation, selective vascular permeability, and formation of an anticoagulant surface (40, 41). Therefore, damage to intercellular tight junctions of endothelial cells increases their permeability; pro-inflammatory factors also induce the expression of adhesion factors on the cell and disrupt the glycocalyx to promote the adhesion and aggregation of leukocytes and platelets (40). In addition, inflammatory mediators trigger apoptotic pathways, and the apoptosis of endothelial cells exposes collagen and further induces platelet adhesion (40). The second mechanism involves shock, hypoperfusion, and microcirculatory microthrombosis, which aggravate ischemia and generate a hypoxic environment for the affected organs and tissues. Under such hypoxic conditions or in response to ischemia/reperfusion injury, damaged cells and mitochondria produce large amounts of ROS and reactive nitrogen species (RNS) (17), which can cause lipid peroxidation, destroy the cell structure, and induce cell death (17, 42). Moreover, ROS/RNS can modify the post-transcriptional regulation of proteins, such as nitrosylation and acetylation, resulting in protein dysfunction, which further affects the function of cells and organs (43). In addition to these common effects, other pathophysiological mechanisms contribute to organ dysfunction in sepsis that vary depending on the cellular composition, tissue structure, and organ function (44).

As shown in **Figure 1**, the existing pattern of clinical presentation as a diagnostic criterion is challenged owing to the heterogeneity of sepsis (45). With the rapid development of molecular biology techniques, the analysis of biological subtypes or endogenous phenotypes of sepsis may be helpful for precise sepsis diagnosis and treatment (46, 47).

## Different Biological Samples in Sepsis Proteomics

Biological samples used in sepsis proteomics are varied and can include body fluids (such as plasma, serum, and urine), tissues or organs (such as the liver, heart, and muscle), cells (such as platelets, lymphocytes, monocytes, neutrophils, and endothelial cells), organelles (mitochondria), and exosomes. Each biological sample has its advantages and disadvantages (48). Plasma and serum are readily available samples in the clinic (49); however, plasma proteomics is the most complex form of proteomics. Plasma proteins include both proteins produced under physiological conditions and proteins secreted and shed by cells or tissues under pathological conditions. There are a wide variety of proteins in plasma, a wide range of sources, and large differences in protein content (49, 50). Among them, the proportion of high-abundance proteins reached approximately 99%, including albumin, IgG, IgA, fibrinogen, transferrin, haptoglobin (HP), and antitrypsin (51, 52). Therefore, during sample preparation, thoroughly removing high-abundant proteins that interfere with the detection and enrichment of low-abundant proteins is key to identifying specific biomarkers for diseases and exploring the potential molecular mechanisms underlying diseases. Currently, there is an increasing number of technologies for the removal of high-abundance proteins from plasma. The classical methods include organic solvent solubilization or precipitation methods and affinity methods. Solvent solubilization and precipitation are consumption methods that reduce the complexity of proteomic samples by removing high-abundance proteins from proteomic samples using organic solvents, such as acetonitrile, ammonium sulfate, and ethanol. Affinity methods include those based on ligands of broad selectivity (e.g., heparin affinity and dye affinity) and those based on antibodies (e.g., immunoaffinity). Among them, the immunoaffinity approaches, which utilize immobilized antibodies for capturing one or more of the high-abundance proteins from biological samples, are the most widely used (51, 53–55). However, there is still no technology that can solve the purification problem for all proteins (56, 57). The current strategy is to use a combination of several different separation and purification techniques for the removal of high-abundance proteins (49, 58, 59). The protein composition in urine is relatively stable compared to that in other body fluids. Although the amount of urinary protein is much lower than that of plasma, more than half of the protein comes from the kidney and urinary tract; therefore, urine proteomics can be used to study the pathophysiological process of kidney disease (60–62). There are proteins secreted in plasma by organs, such as the liver; however, they do not reflect the degree of organ injury (63). Direct analysis of protein expression in organs is therefore beneficial for further understanding the mechanism of organ injury in sepsis. Nonetheless, organs and tissues can only be obtained through animal experiments or autopsies (64). Cytoproteomics is a proteomics technique that uses cells as samples. The cells most closely related to the occurrence and development of sepsis include neutrophils, endothelial cells, platelets, and monocytes. In addition, these cells usually do not include high-abundance proteins (64). The exosome is a bilayer vesicle secreted by cells into the extracellular space that contains proteins, non-coding RNAs, mRNAs, and lipids. It is involved in biological processes, such as immune response, tumor cell migration and invasion, and cell signaling (65–67). Exosomes are present in most body fluids (blood, urine, saliva) and cell culture media (68, 69); additionally, exosomes are one of the sources for screening protein biomarkers (48). Nevertheless, the preparation of exosomes is a complex and time-consuming process (48).

## Proteomics in Basic Research

Because sepsis can easily cause cardiovascular, lung, kidney, liver, and other organ dysfunction—leading to a poor prognosis—in recent years, researchers have begun to focus on the changes in protein expression in various organs in sepsis models and devoted themselves to exploring the molecular mechanism of multiple organ injuries in sepsis. Various animal models of sepsis can currently be induced by surgical (e.g., CLP, cecal ligation and incision, colon ascendant stent peritonitis) or non-surgical means (injection of bacteria or exogenous molecules) to mimic the hemodynamic and immunological changes in human sepsis. The most common of these is the CLP procedure in rodents (48, 64, 70, 71). The results of proteomic studies performed in animal models are summarized in **Table 1**.

**Table 1:** Summary of proteomics studies of sepsis infection using animal models.

Species	Sample	Sepsis Model	Altered Pathways	Proteins	Ref.
Mouse	Kidney	Injected with LPS	Acute phase response	**Upregulated:** Lipocalin-2, Complement C3, Fibrinogen, Haptoglobin and Hemopexin	(72)
Pig	Kidney	Injected with pseudomonas aeruginosa	Endoplasmic reticulum (ER) stress, oxidative stress, mitochondrial energy metabolism, tubular transport, and immune/inflammatory signaling	**Downregulated:** Enoyl-CoA hydratase (cellular energetics), and Chloride intracellular channel protein 4 (transporter) **Upregulated:** 78 kDa glucose-regulated protein (ER stress), Peroxiredoxin-6 (oxidative stress protein), Annexin A1(signal transduction), and Laminin subunit gamma-1(cytoskeleton)	(73)
Rat	Kidney	CLP	Mitochondrial energy production	**Downregulated:** COX5B (48 hours after CLP compared to 24 hours)	(74)
Rat	Brain	CLP	Stress, glycolysis, and mitochondrial energy production	**Downregulated:** Chaperone proteins, Enolase and Glucose-6-phosphate dehydrogenase, Creatine kinase isoenzyme and Aconitase 2, Glutamate oxaloacetate transaminase 1, HSP60 (24 h) Glyceraldehyde-3-phosphate dehydrogenase, Aldolase A (48 h) **Upregulated:** G protein beta 1 subunit and COP9 signalosome complex subunit 4 (48 h)	(75)
Rat	Cerebral cortex	CLP	Coagulation, signaling, immune response, and energy metabolism	**Downregulated:** Glandular kallikrein-10, and Succinate dehydrogenase, **Upregulated:** Alpha-2 macroglobulin, Prolyl endopeptidase, Serine protease inhibitor A3N, Kininogen 2, and Alpha-1-acid glycoprotein	(76)
Mouse	Brain	CLP	Immune and coagulation	**Downregulated:** SMAD4, DPYS, PTGDS **Upregulated:** CUL4A	(77)
Cat	Liver mitochondria	Injected with LPS	Reactive oxygen species production and lipid metabolism	**Downregulated:** HSP70, F1-ATPase and key enzymes regulating lipid metabolism (Acyl-CoA dehydrogenase and HMG-CoA synthase) **Upregulated:** Urea cycle enzymes (Carbamoyl phosphate synthetase-1, Ornithine transcarbamylase), HSP 60 and Manganese superoxide dismutase	(78)
Rat	Hepatic mitochondria	CLP	Mitochondrial functions	**Downregulated:** MP1 and MP2 **Upregulated:** MP3	(79)
Mouse	Liver	CLP	Acute phase proteins, oxidative stress, apoptosis, and nitric oxide metabolism	**Downregulated:** Fibrinogen β precursor (6 h), Carbamoyl phosphate synthetase (24 h) **Upregulated:** Calgranulin B, Cyclophilin A (6 h), and Amyloid formation (12 h)	(80)
Mouse	Lung	CLP	Muscle contraction, oxygen transport, protein synthesis, collagen barrier membranes, cell adhesion, and coagulation function	**Downregulated:** Transferrin **Upregulated:** Myosin light chain 4, Cardiomyopathy associated protein 5, Myoglobin, Hemoglobin subunit, 60S ribosomal protein L6,28,34, Fibrinogen alpha chain, Matrix metalloproteinase-9, Tissue-type plasminogen activator, Semaphorin-7A, OTULIN and MAP3K1	(81)
Rat	Heart	CLP	Mitochondrial function	**Downregulated:** Acyl-CoA synthetase 2-like, 2-oxoglutarate dehydrogenase E1 component, Oxoglutarate dehydrogenase, 2-oxoglutarate dehydrogenase complex, and Succinate Coenzyme A ligase (members of the Krebs cycle)	(82)
Rat	Skeletal muscle	CLP	Oxidative stress and mitochondrial dysfunction	**Downregulated:** HSP60, HSP27, HSP β6, and ATP synthase β-chain **Upregulated:** SOD-1, and Ubiquitin-conjugated proteins	(83)
Pig	Plasma	Peritonitis-induced sepsis was initiated by intraperitoneal injection of autologous feces	Oxidative stress, inflammatory, and cytoskeletal assembly	**Upregulated:** CD14, HP, Hemopexin, Microfilament, Actin filament cross-linker protein isoforms 3/4 and Plectin 1	(84)
Mouse	Plasma	CLP	Inflammation, immunity, and coagulation	**Downregulated:** α-2 HS glycoprotein and Zinca-glycoprotein (metabolism) **Upregulated:** Transferrin, Hemopexin, HP, Serum amyloid protein P, and Kininogen	(85)
Rat	Platelet	CLP	Platelet activation, acute phase proteins, cytoskeleton structure, and energy production	**Downregulated:** Protein disulfide-isomerase associated 3, Glucose-6-phosphate dehydrogenase, and Myosin regulatory light polypeptide 9 **Upregulated:** Fibrinogen gamma chain, Growth factor receptor-bound protein 2, Thrombospondin 1, Alpha-1-antitrypsin precursor, Tubulin alpha 6, ATP synthase beta subunit, and succinate dehydrogenase complex subunit B	(86)

HSP, Heat shock protein; LPS, Lipopolysaccharide; CLP, Cecal ligation and puncture; COX5B, Cytochrome c oxidase subunit B;SMAD4, Mothers against decapentaplegic protein 4; DPYS Dihydropyrimidinase; PTGDS, Prostaglandin-H2 D-isomerase; CUL4A, Upregulating the expression of cullin 4A; OTULIN, Ubiquitin thioesterase; MAP3K1, Mitogen-activated protein kinase kinase kinase 1;Hp, Haptoglobin; SOD-1, Superoxide dismutase-1.

### Renal Proteomics

The kidney is one of the most vulnerable organs in sepsis patients. A total of 22%–51% of sepsis patients admitted in intensive care units experience AKI (87, 88). Understanding the mechanism of AKI in sepsis is therefore essential for the treatment of sepsis patients. The pathophysiological mechanism of AKI in sepsis is unknown. Microvascular dysfunction, inflammation, and metabolic reprogramming are currently considered basic mechanisms of septic AKI (89). Septic AKI still occurs without hemodynamic instability and marked hypoperfusion, even with increased renal blood flow. Inflammatory mediators, including PAMPs and DAMPs released after pathogen invasion, bind to TLRs expressed on immune cells, endothelial cells, and tubular epithelial cells, resulting in endothelial activation, tissue injury, and oxidative stress (89). This series of events ultimately leads to microvascular thrombosis and altered flow continuity (intermittent or no flow) (89). Furthermore, during septic AKI, energy is re-prioritized in the quest to meet vital metabolic needs, prioritizing survival at the expense of cellular function (87, 89, 90). Kellum and Prowle (91) summarized the pathogenesis of AKI caused by various factors. They stated that the main mechanism of AKI in sepsis is an excessive inflammatory response. Róka et al. (72) studied the renal proteome in sepsis and found that renal proteome change was milder in the early phases (1.5 and 6 h) than in the late phases (24 and 48 h). Changes in acute phase proteins (APPs) were the most evident. Of these, lipocalin-2, complement C3, fibrinogen, haptoglobin, and hemopexin were the most upregulated APPs. To further study the molecular mechanism of sepsis-related renal injury, Matejovic et al. (73) selected pigs injected with *Pseudomonas aeruginosa* for kidney biopsy and dynamic proteomics analysis. Twenty differentially expressed proteins were distinguished between the sepsis and control groups. Their study showed that endoplasmic reticulum stress (78 kDa glucose-regulated protein), oxidative stress (peroxiredoxin-6), mitochondrial energy metabolism (enoyl-CoA hydratase), tubular transport (chloride intracellular channel protein 4), and immune/inflammatory signaling (annexin A1, and laminin subunit gamma-1) are activation pathways of AKI in sepsis. Hinkelbein et al. (74) performed proteomic analysis in renal tissues from healthy rats and rats with CLP sepsis (24 and 48 h following CLP). The expression of cytochrome c oxidase subunit B (COX5B) was lower in the 48-h sepsis group than in the control and 24-h groups. Cytochrome oxidase (Complex IV) is an enzyme located at the end of the electron transport chain in the inner mitochondrial membrane and functions as a proton pump (92). Therefore, it is speculated that the occurrence of renal injury in sepsis is closely related to mitochondrial dysfunction. It should be mentioned that different studies yielded different results, possibly resulting from different experimental models, experimental animals, and timing (early and late phases in sepsis).

### Cerebral Proteomics

Sepsis-associated encephalopathy (SAE) is defined as diffuse brain dysfunction secondary to systemic infection (93). The manifestations are varied but lack specificity. SAE can present as delirium, agitation, and changes in consciousness (94). More than half of sepsis patients will show features of SAE (93); it can often be the first symptom of sepsis and may lead to long-term impairment (94, 95). Hinkelbein et al. (75) investigated the changes in brain protein expression over time in rats after sepsis induction. Twenty-four proteins were downregulated—including those involved in stress (chaperone proteins), glycolysis (enolase and glucose-6-phosphate dehydrogenase), and mitochondrial energy production (creatine kinase isoenzyme and aconitase 2)—24 h after sepsis induction. After 48 h, 2 proteins were upregulated (G protein beta 1 subunit and COP9 signalosome complex subunit 4), and 3 metabolism-related proteins were downregulated (glutamate oxaloacetate transaminase 1, glyceraldehyde-3-phosphate dehydrogenase, and aldolase A). Glycolysis-related enzyme production is reduced and mitochondrial function impaired, ultimately leading to impaired energy production in brain cells. In addition, chaperonin 60 (HSP60) was found to be downregulated in their study. HSP is a protein generated by cells in response to the induction of stressors (e.g., heat, infection, poisoning, trauma, etc.), and it improves the stress capacity of cells to protect them from the deleterious effects of an imbalance in proteostasis (96, 97). It has been shown that HSP60 also has a neuroprotective effect (98). Therefore, Hinkelbein et al. (75) suggested that the downregulation of HSP60—one of the mechanisms causing brain dysfunction in sepsis—makes brain cells more vulnerable to stress. Yang et al. (76) used isobaric tags for relative and absolute quantification (iTRAQ) technology to study the cerebral cortex of sepsis rat models and identified 91 differentially expressed proteins. These proteins are related to signaling (e.g., succinate dehydrogenase), energy metabolism (e.g., serine protease inhibitor A3N), coagulation (e.g., kininogen 2), and immune response (e.g., alpha-1-acid glycoprotein). Among them, alpha-2 macroglobulin and kininogen, the expression of which was upregulated, act on the complement and coagulation systems, inhibit coagulation, and enhance immunity. The upregulation of prolyl endopeptidase, which hydrolyzes multiple polypeptide neurotransmitters and hormones, may lead to cognitive dysfunction in patients with SAE. The expression of glandular kallikrein-10, involved in the formation of the kallikrein-kinin system, was substantially downregulated, which may be responsible for insufficient blood supply to the brain and increased apoptosis of brain cells in patients with sepsis (76). Xie et al. (77) applied quantitative proteomics based on iTRAQ to analyze the therapeutic mechanism of 2% hydrogen inhalation on brain injury in a sepsis mouse model. A total of 39 differentially expressed proteins were identified in the study, and the functions and pathways of all proteins were analyzed using Gene Ontology (GO) functions and the Kyoto Encyclopedia of Genes and Genomes (KEGG). It was found that H_2_ played a role in regulating the immune system and coagulation system. Thus, the protective mechanism of H_2_ on SAE was revealed. H_2_ decreased SAE in septic mice by downregulating the protein expression of *Drosophila* mothers against decapentaplegic protein 4 (SMAD4), dihydropyrimidinase (DPYS), and prostaglandin-H_2_ D-isomerase (PTGDS), and upregulating the expression of cullin4A (CUL4A) (77).

### Liver Proteomics

Wang et al. (78) demonstrated that exosomes released from LPS-induced macrophages contain several proinflammatory factors involved in the process of sepsis-induced acute liver injury by regulating the activation of multiple inflammatory pathways (e.g., the NLRP3 inflammasome pathway). It has also been reported that heat shock-induced exosomes from hepatocytes promote liver injury by activating NOD-like receptor signaling pathways (99). Liver mitochondrial proteomics analysis showed altered protein patterns associated with important metabolic pathways, such as regulating ROS production and lipid metabolism, during acute endotoxemia (100). The expression of urea cycle enzymes (carbamoyl phosphate synthetase-1 and ornithine transcarbamylase), HSP 60, and manganese superoxide dismutase increased, whereas the expression of HSP70, F1-ATPase, and key enzymes regulating lipid metabolism (acyl-CoA dehydrogenase and HMG-CoA synthase) was decreased. Chen et al. (79) investigated the changes in liver mitochondrial proteome during sepsis and the role of heat shock treatment. The heat-shock treatment model requires to heat the whole body 24 h before CLP surgery after anesthetizing the rats. Rectal temperature was maintained between 41°C and 42°C for 15 min. After heat shock treatment, HSP-72 was increased in the cytoplasm of rat livers. Three variants (MP1, MP2, and MP3) of aldehyde dehydrogenase 2 (ALDH2) were detected in rat liver mitochondria using two-dimensional gel electrophoresis (2D-GE) separation and liquid chromatography with tandem mass spectrometry (LC-MS/MS) analysis. The expression of MP1 and MP2 was downregulated, whereas that of MP3 was upregulated in the early (9 h after CLP) and late (18 h after CLP) phases of sepsis. However, heat shock treatment reversed this effect. In addition, ALDH2 activity was reduced during sepsis, especially in the late phase of sepsis, as shown by an enzyme activity assay. In contrast, heat shock treatment contributed to the retention of ALDH2 activity. ALDH2 is an important oxidative stress factor *in vivo*. Studies have shown that its overexpression or increased activity can effectively promote the metabolism of toxic aldehydes, inhibit mitochondrial damage, and play an important role in various diseases (101–103). Dear et al. applied the 2D difference in gel electrophoresis (2D-DIGE) technique to study sepsis-induced early (6 h after CLP vs 6 h after sham) and late (24 h after CLP vs 6 h after CLP) proteomic changes in the liver and verified their abundance changes using western blotting. At 6 h after CLP, the protein with the greatest increase in abundance was calgranulin B and that with the greatest decrease was fibrinogen β precursor. At 24 h after surgery, the protein with the greatest increase was associated with amyloid formation, and the greatest decrease was observed in carbamoyl phosphate synthetase. These and other proteins with altered abundance are involved in processes such as acute phase response, oxidative stress, apoptosis, and nitric oxide (NO) metabolism. One of these proteins, cyclophilin A, increased significantly at 6 h after CLP. Cyclophilin, one of the most abundant proteins in the cytoplasm, is involved in various cellular pathways, including immune regulation, cell signaling, transcriptional regulation, and protein folding and trafficking (104, 105). Notably, cyclophilin interacts with the extracellular receptor CD147 and plays an important role in the regulation of inflammatory responses in a variety of diseases (106). This group found that sepsis-induced renal injury was reduced when CD147 was inhibited, along with a significant reduction in serum cytokine production. Notably, however, the inhibition of CD147 did not significantly reduce sepsis-induced liver injury, as determined by measuring AST and ALT levels to indicate the degree of liver injury. The authors speculated that this may be owing to different pathways of liver and kidney injury in sepsis (80).

### Lung Proteomics

ARDS is a clinical syndrome caused by intrapulmonary or extrapulmonary sources characterized by refractory hypoxemia. In ARDS, the common intrapulmonary and extrapulmonary causes are pneumonia and sepsis, respectively (107–109). It has been shown that 2% hydrogen can effectively ameliorate multiple organ damage and increase the survival rate of sepsis mice (110–112). Bian et al. (81) identified differentially expressed proteins, and then elucidated the molecular mechanism of H_2_ in treating acute lung injury in sepsis using iTRAQ-based quantitative proteomics analysis. In this study, through functional enrichment analysis, the identified differentially expressed proteins were classified according to their functions, which included muscle contraction (myosin light chain 4, cardiomyopathy associated protein 5), oxygen transport (myoglobin and hemoglobin subunit), protein synthesis (60S ribosomal protein L6,28,34), collagen barrier membrane (matrix metalloproteinase-9), cell adhesion (Semaphorin-7A), and coagulation (fibrinogen alpha chain and tissue-type plasminogen activator). In addition, the expression of Semaphorin-7A, OTULIN, and MAP3K1 increased in sepsis, whereas that of transferrin decreased. H_2_ alleviated acute lung injury in septic mice by downregulating Semaphorin-7A, OTULIN, and MAP3K1 expression and upregulating transferrin expression. Thus, it was demonstrated that the protective effect of H_2_ on sepsis-related lung injury may be due to an improvement in the oxygen transport capacity of sepsis mice by alleviating mitochondrial injury and the abnormal metabolism of skeletal muscle. This leads to the strengthened contraction of the diaphragm and limb skeletal muscles, improving respiration and circulation.

### Heart Proteomics

In patients with septic shock, the incidence of hypofunction is approximately 60% (113), with a high mortality rate. Some studies have applied 2D-GE, MS, and ingenuity pathway analysis to determine the changes in protein levels between sepsis and non-sepsis states (82). They found that 12 proteins were significantly altered in the heart. Among the cardiac-related differentially expressed proteins, five (acyl-CoA synthetase 2-like, 2-oxoglutarate dehydrogenase E1 component, oxoglutarate dehydrogenase, 2-oxoglutarate dehydrogenase complex, and succinate coenzyme A ligase) are members of the Krebs cycle and their expression was downregulated 48 h after sepsis induction. These proteins were associated with impaired energy production in the heart. Numerous studies have shown that mitochondrial reduction can cause a decrease in cardiac function in sepsis (114). Therefore, in sepsis, energy failure is an important pathophysiological mechanism leading to septic cardiomyopathy (115).

### Skeletal Muscle Proteomics

In sepsis, proteins are in a hypercatabolic state (decreased synthesis and increased degradation), leading to substantial muscle atrophy. Using a model of burn-related sepsis, Duan et al. (83) identified differentially expressed proteins for muscle atrophy in sepsis. The burn-related sepsis model was established by performing CLP surgery 2 days later in animals with full-thickness skin burns reaching 20% of the total surface area. In their study, some chaperone proteins (HSP60, HSP27, and HSP β6) and metabolic enzymes (ATP synthase β-chain) were downregulated, while SOD-1 expression was upregulated. HSPs have a role in preventing oxidative stress, assisting protein synthesis, and repairing misfolded proteins. Thus, the downregulation of HPS exacerbates oxidative stress-induced proteolysis. The downregulation of metabolic enzyme expression may reduce cellular energy production, which may hinder protein translation (a process that requires ATP). These results suggest that oxidative stress and mitochondrial dysfunction play an essential role in sepsis-induced skeletal muscle atrophy. Duan et al. (83) also reported increased levels of ubiquitin-conjugated proteins (E2) in the muscle of rats with burn-related sepsis, suggesting that the ubiquitin-proteasome pathway is pivotal to protein metabolism in skeletal muscle. Proteins targeted for degradation *via* this pathway are first labeled by ubiquitin molecules mediated by ubiquitin-related enzymes (E1, E2, and E3), that is, ubiquitination of proteins, and later proteolyzed by the proteasome (116). Mass spectrometry can be used for the ubiquitination analysis of biological samples, which suggests the research prospect of proteomics in this direction.

### Plasma Proteomics

Most current research techniques in plasma proteomics can remove highly abundant proteins. Nonetheless, there is no efficient method for the removal of moderately abundant proteins, and this is the key to the detection and quantification of low-abundant proteins (117). Thongboonkerd et al. (84) first used a large animal model to study the changes in the plasma proteome of sepsis. In this study, differential proteomics indicated altered plasma levels of 36 proteins (30 upregulated and 6 downregulated) representing 27 unique proteins. Among them, plasma CD14, HP (acute-phase reaction proteins and involved in oxidative stress pathways), and hemopexin (an anti-inflammatory molecule and an oxidative scavenger) were increased in early sepsis. In addition, levels of microfilament, actin filament cross-linker protein isoforms 3 and 4, and plectin 1, which are involved in cytoskeletal assembly, were also increased. Ren et al. (85) compared differentially expressed proteins in the plasma of CLP-operated and sham-operated mice using MS and western blotting after isolating plasma proteins. Plasma protein changes were observed at 4 h (early phase) and 24 h (late phase) after CLP. They demonstrated that significant changes in plasma proteins occurred at 24 h, but not at 4 h after surgery. The identified differentially expressed proteins were associated with inflammation, immunity, and coagulation. The findings suggest that the plasma abundance of fibrinogen and several plasma protease inhibitors (serine/cysteine proteinase inhibitor) change in sepsis, emphasizing the interaction between the inflammatory response and altered coagulability. In addition, the upregulated levels of proteins involved in heme and iron metabolism (e.g., transferrin, hemopexin, HP) confirmed that iron treatment played an important role in innate immune activation. They also found downregulation of two proteins involved in metabolism, the a-2 HS glycoprotein (Fetuin A) and zinc-α glycoprotein.

In summary, in the proteomics of various organs during sepsis, the main pathway affected is the energy generation pathway, especially mitochondrial disorders. The downregulation of mitochondria-related proteins was found in almost all organ dysfunctions. This conclusion is consistent with that of Hohn et al. (118). In their study, differentially expressed proteins were obtained *via* dynamic studies (12, 24, and 48 h) of the organ proteomes and serum proteomes. Separate network analysis of these proteins revealed that changes in the sepsis organ proteome were related to redox activity, cellular energy production and metabolism, and nucleotide or nucleoside metabolism, while those in the plasma proteome were related to lipoprotein metabolism, coagulation, and inflammation.

## Advantages and Disadvantages of Animal Models

Laboratory animals have a similar genetic inheritance and can be easily controlled for body weight and age. These factors increase the comparability of experiments but do not reflect the heterogeneity of humans (48). At the same time, this allows researchers to avoid the risks caused by experiments on humans, and researchers can obtain different samples according to the purpose of the study and even sacrifice animals. However, animal models cannot fully reproduce the complexity of human sepsis (119). Furthermore, there is no physiological monitoring in animal models, and the severity of sepsis can only be roughly estimated based on the time of death and mortality (120). The above limitations make the experimental results obtained from animal models not fully applicable in clinical practice.

## Proteomics in Clinical Studies

Although sepsis can occur at all ages, sepsis in the elderly and neonates is characterized by a high incidence and high mortality (121–123). Neonates (especially premature infants) have an immature immune system, poor immune function, and reduced immune function with age (124). Sixty percent of patients with sepsis are elderly (aged ≥65 years), and this proportion may increase with an aging population (125). It has been shown that there are differences in the proteomes of sepsis patients of different ages (126). Cao et al. (126) collected plasma from community-acquired pneumonia (CAP) patients aged 50–65 years and 70–85 years, with and without sepsis, as samples for semi-quantitative plasma proteomics. Fifty-eight proteins were identified, whose expression correlated with age. These proteins were involved in acute phase response (e.g., C-reactive protein, lipopolysaccharide binding-protein, α-1-antichymotrypsin, and transthyretin [TTR]), coagulation pathway (e.g., fibrinogen α chain, fibrinogen β chain, fibrinogen γ chain, and VWF), lipid metabolism (e.g., apolipoprotein B-100 [Apo B100], Apo C, and Apo E), atherosclerosis signaling (e.g., Apo B-100, Apo C, Apo E), and NO and ROS production (e.g., lysozyme C, and clusterin). Therefore, we next discuss the research progress of proteomics in neonatal and adult sepsis. The proteomics results in these clinical studies are summarized in **Table 2**.

**Table 2:** Summary of proteomics of sepsis infection in clinical studies.

Experimental Plan	Sample	Altered Pathways	Proteins	Ref
39 CAP patients (50−65 and 70−85 years old) who did or did not develop severe sepsis	Plasma	Acute phase response, coagulation pathway, lipid metabolism, atherosclerosis signaling, and production of nitric oxide and reactive oxygen species	**Downregulated:** TTR, Apo C III, and Clusterin, **Upregulated:** CRP, LBP, A1ACT, A1AG, Fibrinogen α/β/γ chain, VWF, Apo B-100, Apo E, and Lysozyme C	(126)
Patients who delivered preterm and had intra-amniotic inflammation in response to infection *versus* patients who had symptoms of preterm labor but delivered at term	Amniotic fluid	——	MR score: Neutrophil defensins-1 and-2, Calgranulins A and C	(127)
Newborns with culture-confirmed EONS *versus* gestational age -matched controls	Cord blood	Transfer/carrier, immunity/defense, and protease/extracellular matrix	**Downregulated:** Albumin, Apo A4, Apo E and Apo H **Upregulated:** HP, HpRP, AFP and VDBP	(128)
Survivors *versus* non-survivors on day 28 in patients with sepsis and septic shock	Serum	Complement replacement pathway and acute phase response	Complement factor B subunit Bb, HP, and Clusterin were more significantly upregulated in survivors α-1-B-Glycoprotein was upregulated to a greater extent in non-survivors than in survivors	(129)
Adult male patients diagnosed with sepsis (non-survivors and survivors = 6 each)	Serum	α1 globulins, α2 globulins, and danger−associated molecular patterns/Alarmins	Hp, TTR, ORM1, A1AT, SAA and S100A9 exhibited differential expression patterns between survivors and non-survivors	(130)
Septic patients secondary to CAP *versus* age- and gender-matched healthy volunteers	Peripheral blood mononuclear cells (PBMC) and polymorphonuclear cells (PMN)	Alterations in cytoskeleton, cellular assembly, movement, lipid metabolism, and immune responses in septic patients	**Downregulated:** Apolipoprotein family proteins; F2, GSN and PON1 (inflammation, and coagulation); PLEC, GCC2(cytoskeleton and cell motility) **Upregulated:** HP, FGG, ATM, SERPINA1, SERPINA3, CRP and LBP (inflammation, and coagulation); KIF27, NF1, MYH9, MYO5B, ALMS1, SYNE1, ASPN (cytoskeleton and cell motility)	(131)
Sepsis secondary to HAP *versus* healthy volunteers	Plasma	Lipid metabolism	**Downregulated:** PON1, Apo A1, Apo C, and Apo E **Upregulated:** HP, and SAA1/SAA2	(132)
15 sepsis and 15 SIRS patients	Urine	Inflammation, immunity, and structural or cytoskeletal processes	**Downregulated:** Complement 3, SERPINA1, and Ceruloplasmin **Upregulated:** Cadherin 1, and HP	(133)
Survivors *versus* non-survivors on day 28 in patients with sepsis	Urine	Biological processes of lipid homeostasis, cartilage development, iron ion transport, and certain metabolic processes	**Downregulated:** LAMP-1 and DPP-4 (non-survival) **Upregulated:** SELENBP-1, HSPG-2, A-1-BG, HPR, and LCN (non-survival)	(134)
Septic patients and matched healthy controls	Platelets	Inflammatory response and coagulation activation	**Upregulated:** EFCAB7 (calcium ion binding), Actin (cytoskeleton), IL-1β (cytokine), GPIX (membrane receptor), and GPIIb (integrin)	(135)
Patients with septic shock	Monocytes	Immune response and energy metabolism	**Downregulated:** Oxidative phosphorylation and the Krebs cycle, β-oxidation of fatty acids, the related interferon signaling pathway, MHC II antigen presentation pathway **Upregulated:** Glycolytic metabolism	(136)

CAP, Community-acquired pneumonia; TTR, Transthyretin; Apo, Apolipoprotein;LBP, Lipopolysaccharide binding protein; A1ACT, a-1-antichymotrypsin; A1AG a-1-acid glycoprotein; MR, Mass Restricted; CRP, C-reactive protein; EONS early-onset neonatal sepsis; HpRP, Haptoglobin-related protein; AFP, a-fetoprotein; VDBP,Vitamin-D binding-protein; GSN, a1-antitrypsin, A1T1/SERPINA; serum amyloid A, SAA; orosomucoid 1/a1 acid glycoprotein, ORM1; Prothrombin, F2; Gelsolin; PON1, Paraoxonase 1; PLEC, Plectin; GCC2, GRIP and coiledcoil domain-containing protein 2; FGG, Fibrinogen gamma chain; KIF27, Kinesin-like protein KIF27; NFI, Neurofibromin; MYH9, Myosin-9;MYO5B, Unconventional myosin-Vb; ALMS1, Alstrom syndrome protein 1;SYNE1, Nesprin-1;DPP-4, Dipeptidyl peptidase-4; HPR/HP, Haptoglobin; EFCAB7, EF-hand calcium-binding domain-containing protein 7; IL,Interleukin; GPIX, Glycoprotein IX; GPIIb, Glycoprotein IIB; MHC, Major histocompatibility complex; HLA, Human leukocyte antigen.

### Neonatal Sepsis Proteomics

Neonatal sepsis is divided into early and late-onset according to the time of symptom onset. Early-onset often appears within 72 h of birth and usually results from vertical transmission from mother to child. The late-onset form appears 3–7 days after birth and is usually caused by surrounding environmental factors (137–139). At different gestational weeks, the composition of amniotic fluid has few similarities and can be categorized into maternal serum dialysis fluid (early pregnancy), fetal urine (mid pregnancy), and pulmonary secretions (late pregnancy). The fetus swallows amniotic fluid throughout the pregnancy and is therefore directly involved in changes in amniotic fluid material (140). In addition, neutrophils in amniotic fluid are partially derived from the fetus, and assessment of the amniotic fluid reflects the fetal inflammatory response to infection (141, 142). Therefore, proteome analysis in amniotic fluid provides new hints for the diagnosis and prevention of neonatal sepsis. Surface-enhanced laser desorption/ionization time-of-flight MS (SELDI-TOF-MS) is a proteomics technology that combines chromatography with MS, and the analysis is based on a specific algorithm developed for retrieving information on clinically and biologically relevant biomarkers from proteomic SELDI tracing (143). First, samples to be examined are added to a protein array on the chip surface. The protein molecules bind to the chip according to their specific biological or physicochemical properties (hydrophilicity, hydrophobicity, ion exchange, and metal binding), which also facilitate their capture, retention, and purification. Unbound or non-specifically bound proteins are washed out to obtain only specific bound molecules. Finally, a chromatogram is generated using TOF-MS for identification (144, 145). SELDI-TOF-MS enables the use of extremely small amounts of raw biological fluids and rapid screening of numerous biological samples simultaneously (146). Buhimschi et al. (127) performed amniocentesis in 169 women with a singleton pregnancy who gave birth prematurely or with fetal membranes. The protein fingerprint of amniotic fluid was obtained using SELDI-TOF-MS and quantified using the mass restricted (MR) score. The MR score includes the expression of four proteins: neutrophil defensin-1 and-2 and calgranulin A and C (64, 147). It is used to identify amniotic inflammation and is associated with tissue chorioamnionitis and early-onset neonatal sepsis(EONS) (148). Abnormal MR scores strongly correlate with early-onset sepsis, as demonstrated by Buhimschi et al. Calgranulin A and C in amniotic fluid are associated with early-onset sepsis and neurodysplasia in neonates (148). Despite the many advantages of MR scoring, amniotic fluid is not readily available, and the functional protein network associated with early-onset sepsis cannot be directly observed (128). Proteomic analysis of cord blood was therefore performed by Buhimschi et al. (128). In their study, 19 differentially expressed proteins were identified in the cord blood of EONS cases using 2D-DIGE and MS. These proteins were classified as transfer/carrier, immunity/defense, and protease/extracellular matrix according to Ontological classifications. HP, haptoglobin-related protein (HpRP), a-fetoprotein (AFP) and vitamin-D binding-protein (VDBP) (upregulated) and albumin, Apo A4, Apo E, and Apo H (downregulated) are synthesized by hepatic parenchymal cells. Thus, the liver is an important organ mediating inflammatory and immune responses in EONS. In addition, Buhimschi et al. (128) confirmed using western blotting that the Hp and HpRP lanes were evident in cord blood of EONS (“switch-on”) but not in neonates with non-early-onset sepsis (“switch-off”). Therefore, the HP “switch-on” pattern may become a biomarker of early sepsis in preterm infants.

### Adult Sepsis Proteomics

Kalenka et al. (129) also compared the serum protein of survivors and non-survivors of sepsis or septic shock and identified six differentially expressed proteins (Complement factor B subunit Bb, α-1-B-glycoprotein, HP, and clusterin). These proteins are involved in the complement replacement pathway and acute phase response; they are part of the inflammatory and cytoprotective signaling pathways. To the best of our knowledge, this is the first serum proteomic analysis of patients with sepsis and septic shock to discover several proteins differentially expressed in survivors and non-survivors. Kalenka et al. (129) also demonstrated that proteomics is a feasible tool to identify early alterations in protein expression in patients with sepsis. In a prospective observational study, serum proteome changes from early to late stages were analyzed in sepsis survivors *versus* non-survivors (130). The study identified differences in the levels of HP (acute phase protein), TTR (negative acute phase protein), orosomucoid 1/α1 acid glycoprotein (ORM1, acute phase protein), α1 antitrypsin (A1AT, complement and coagulation pathways), serum amyloid A (SAA), and S100A9 (regulation of inflammatory processes and immune response) between survivors and non-survivors, especially in the early stages of sepsis. Thus, in non−survivors, a dysregulated inflammatory response may be responsible for the death. Sharma et al. (131) selected sepsis patients secondary to CAP as the study subjects to avoid patient heterogeneity and previous interventions. Proteins from sepsis patients were compared with those from age- and sex-matched healthy volunteers. Bioinformatics analysis of the differentially expressed proteins showed that proteins related to the cytoskeleton and cell motility, lipid metabolism and immune response, and other related processes were altered in patients with sepsis. Proteins related to the cytoskeleton and cell motility include those of cell assembly, such as KIF27, NF1, MYH9, MYO5B, ALMS1, SYNE1, and ASPN (upregulated); GSN, PLEC, PON1, F2, and GCC2 (downregulated); and a dynein heavy chain family member (a microtubule-dependent motor ATPase). The apolipoprotein family proteins are downregulated, such as Apo A1, Apo A2, Apo A4, Apo B, Apo C1, Apo C2, Apo C3, and Apo E. Proteins related to inflammation and coagulation include HP, FGG, ATM, SERPINA1, SERPINA3, CRP, and LBP (upregulated) and F2, GSN, and paraoxonase 1 (PON1) (downregulated). In addition, a higher expression of gelsolin and depletion of actin was observed in surviving patients. In a further assessment of lipids and lipoproteins in plasma, it was found that the total cholesterol, HDL-C, and Apo A-I levels were remarkably reduced in septic plasma (131). These results reveal that alterations in cellular structure and lipid metabolism in patients with may be the target for future interventions (131). Another study on plasma proteomics in sepsis patients secondary to hospital-acquired pneumonia (HAP) showed that impaired lipid metabolism was an important alteration in sepsis patients (132). Decreased expression of PON1 and apolipoproteins (Apo A1, Apo C, and Apo E) associated with HDL and increased expression of HP and SAA1/SAA2 were observed. The validation trial indicated that the total plasma cholesterol, HDL-C, LDL-C, non-HDL cholesterol, apolipoproteins ApoA1 and ApoB100, and PON1 levels were downregulated in patients with HAP. These results are similar to the changes in septic patients secondary to CAP and are consistent with the literature emphasizing the important role of lipid metabolism in the pathogenesis of sepsis (132).

Su et al. (133) identified 34 differentially expressed proteins using iTRAQ labeling and 2D-LC-MS analysis of urine from patients with sepsis and systemic inflammatory response syndrome. GO and KEGG analyses indicated that these differentially expressed proteins were involved in inflammatory, immune, and cytoskeletal processes. Among them, five specific proteins were selected by a protein-protein interaction network, which are cadherin 1 (involved in actin cytoskeletal alterations), HP (an anti-inflammatory agent), complement 3, SERPINA1 (inflammatory), and ceruloplasmin (antioxidant and anti-inflammatory defense). The same group also published another article (134), in which proteomic and bioinformatic analyses of urine from sepsis patients with different prognoses (non-survival and survival) revealed that 5 proteins were upregulated (SELENBP-1, HSPG-2, A-1-BG, HPR, and LCN) and 2 proteins were downregulated (LAMP-1 and DPP-4) in the non-survival sepsis group. Three differentially expressed proteins (LAMP-1, SBP-1, and HSPG-2) that had not been reported were validated using western immunoblotting. In non-survivors, LAMP-1 expression was significantly reduced, whereas SBP-1 and HSPG-2 expression did not differ between the survivor and non-survivor groups; thus, urinary LAMP-1 level may be considered to evaluate sepsis prognosis (134). The inflammatory response and activation of coagulation are two important responses of the host defense system in sepsis (149). The coagulation response generated by inflammation induction, in turn, promotes inflammation, and the two interact to form a positive feedback network that promotes the exacerbation of sepsis (149, 150). Neutrophils, monocytes, macrophages, platelets, and other cells play an important role in the development of sepsis. Platelets are enucleated cells, and proteomics can therefore be applied to identify changes in platelet proteins in sepsis. Liu et al. (135) applied 2-DE and MALDI-TOF-MS to identify platelet-derived differentially expressed proteins between sepsis patients and matched healthy controls. This study showed that sepsis patients have increased expression of five platelet proteins: EFCAB7 (calcium ion binding), actin (cytoskeleton), IL-1β (cytokine), GPIX (membrane receptor), and GPIIb (integrin) (135). These five proteins are involved in inflammatory response and coagulation activation, emphasizing the important role of platelets in sepsis-induced inflammation and coagulation. In contrast, in rats, Hu et al. measured the changes in platelet protein expression 12–24 h after the onset of sepsis to document the response of the platelet proteome to sepsis (86). In this study, the expression of eight proteins increased and the expression of four proteins decreased in platelets from the sepsis group compared with those from the control group. These 12 proteins were divided into the following four categories based on the biological system: 1) platelet activation (fibrinogen gamma chain, growth factor receptor-bound protein 2, and thrombospondin 1); 2) acute phase proteins (protein disulfide-isomerase associated 3, alpha-1-antitrypsin precursor, and thioredoxin); 3) cytoskeletal structure (myosin regulatory light polypeptide 9 and tubulin alpha 6); and 4) energy production (ATP synthase beta subunit, glucose-6-phosphate dehydrogenase, and succinate dehydrogenase complex subunit B). Zhang et al. (151) applied iTRAQ quantitative proteomics to study changes in the proteome of monocyte membranes before and after LPS treatment. A total of 1651 proteins were identified, and subcellular analysis of these proteins indicated that more than 90% of mitochondrial membrane proteins were significantly downregulated. This result demonstrates that the mitochondria may be the main target of bacterial infection in sepsis. Zhang observed that the antigen presentation molecules MHC I and MHC II responded differently to LPS treatment. MHC II molecules (CD74 and HLA-DR) were downregulated, whereas MHC I molecules (HLA-A, -B, and -C) were upregulated. De Azambuja Rodrigues et al. (136) applied LC-MS/MS to identify monocyte proteins from patients with septic shock. The downregulated proteins in sepsis have been implicated in oxidative phosphorylation and the Krebs cycle (ATP5C1, DLST, ETFB, NDUFA11 NDUFA2, NDUFS7, NDUFS8, PDK3, PDP1, PDPR, RXRA, SUCLG2, TACO1, and UQCRQ), β-oxidation of fatty acids (ACADM, DECR1, PCCA, and PCCB), the related interferon signaling pathway (EIF2AK2, EIF4A3, EIF4E2, HLA-DPA1, HLA-DQA2, HLA-DRA, HLA-DRB1, IFIT1, MX1, NUP35, OAS3, PSMB8, and UBE2L6), and MHC II antigen presentation pathway (CD74, CTSH, DCTN3, DYNC1LI2, HLA-DMA, HLA-DMB, HLA- DPA1, HLA-DQA2, HLA-DRA, HLA-DRB1, KIF2A, and OSBPL1A). The upregulated proteins were related to glycolytic metabolism (canonical enzymes PGK1, ALDOA, ALDOC, GADPH, PKLR GPI, and LDHA). The above proteomic results suggest that patients with septic shock show disturbances in monocyte immune response and energy metabolism. The studies by Zhang and De Azambuja Rodrigues suggest impaired monocyte antigen presentation in sepsis.

## Disadvantages of Human Samples

Human samples are remarkably heterogeneous. The clinical symptoms and rate of progression of sepsis can vary widely among people; thus, sample collection may occur at various stages of disease progression. Secondly, proteomics studies of human tissues must be performed on post-mortem or biopsy samples. Therefore, many studies prefer to use animal experiments as the first step (64).

## Important PTMs of Proteins

PTM refers to the chemical modification of proteins after translation, regulating the activity, localization, folding, and interaction between proteins and other biological macromolecules (including nucleic acids, proteins, and lipids) (152). Several studies have found that many important life activities and disease occurrence are not only correlated with the abundance of proteins but, more importantly, regulated by PTMs of various types of proteins. Through an in-depth study of the differences in the changes in PTMs, it is important to reveal the pathogenesis of diseases, screen clinical markers of diseases, and identify the targets of drugs. These typically include phosphorylation, glycosylation, ubiquitination, nitrosylation, methylation, acetylation, lipidation, and proteolysis. There are increasing studies on various protein modification omics to elucidate these PTMs. These PTM omics are often combined with proteomics to facilitate more in-depth studies of pathophysiological mechanisms or the development of new biomarkers. For example, the combined application of proteomics and phosphoproteomics can more truly reflect the relationship between protein kinases and substrates. Phosphorylation modification can be different in these proteins whose expression is not different (153). The studies of PTMs in sepsis are summarized in **Table 3**.

**Table 3:** Summary of post-translational modifications in sepsis.

Protein	Type of PTM	Main Conclusions	Ref.
ALDH2	Phosphorylation	MP1 and MP2 with higher phosphorylation have a higher enzymatic activity than MP3 with lower phosphorylation (MP1, MP2 and MP3 are the subtypes of ALDH2).	(79)
HMGB1	Redox modification Acetylation/deacetylation	ROS partially oxidize HMGB1 to form disulfate-type HMGB1, inducing inflammatory cells to produce a range of cytokines to promote the inflammatory response. Lys acetylation after stimulation with LPS and TNF-α, leads to conformational changes in HMGB1, separation from SIRT1 and transport to the cytoplasm, followed by the release into the extracellular space, which would subsequently activate downstream inflammatory signaling.	(43, 154, 155)
Histone H3	Citrullination	CitH3 was significantly increased in the CLP-induced mice sepsis model. Inhibitors of PAD4 modulate citrullination and reduce CitH3 levels, thereby improving survival in sepsis mice.	(156, 157)
NLRP3	Phosphorylation Ubiquitylation	Phosphorylation of serine 5 in the PYD inhibits the activation of NLRP3. Sequential ubiquitination of NLRP3 by RNF125 and Cbl-b inhibits the activation of NLRP3 and prevents the development of sepsis in mice.	(158, 159)
Cysteine residues of various signaling proteins	Nitrosylation	SIRT1 activity decreases with increasing S-nitrosylation of SIRT1, resulting in extracellular HMGB1 release. Increased levels of iNOS, leading to enhanced production of NO, induces S-nitrosylation of SIRT1. During the transformation of LPS-stimulated macrophages from the resting state to the M1 type, the expression levels of iNOS and NO were increased, and mitochondrial complexes I and IV were modified by nitrosylation, resulting in the destruction of the mitochondrial electron transfer chain and inhibition of OXPHOS.	(160–162)

ALDH2, Aldehyde dehydrogenase; HMGB 1, High mobility group box 1; ROS, Reactive oxygen species; LPS, Lipopolysaccharide;TNF, Tumor necrosis factor, SIRT1, Sirtuin1; CitH3, Citrullinated Histone H3; CLP, Cecal ligation and puncture; PAD4, Protein-arginine deiminase type-4; NLRP3, NOD-like receptor protein 3; PYD, Pyrin domain;iNOS, inducible nitric oxide synthase; NO, Nitric oxide; OXPHOS, Oxidative phosphorylation.

In a study conducted by Chen et al. (79), liver mitochondrial proteins in CLP rats were isolated and evaluated using two-dimensional gel electrophoresis, and the protein spots were visualized using silver staining and analyzed with Bio-2D software. Three spots of the same molecular weight (MP1, MP2, and MP3) were significantly altered (79). All three spots are the same enzyme, ALDH2. During sepsis, MP1 and MP2 are downregulated, whereas MP3 is upregulated concomitantly (MP1 and MP2 shift to MP3), leading to a decrease in the ALDH2 activity. In addition, MP1 and MP2 presented a higher degree of protein phosphorylation than MP3. Thus, it is speculated that protein phosphorylation may affect ALDH2 enzymatic activity; that is, MP1 and MP2 with higher phosphorylation have a higher enzymatic activity than MP3 with lower phosphorylation (79). This study demonstrated that a PTM, phosphorylation of ALDH2, may play a role in the pathogenesis of sepsis and provide a new target for the therapy (79).

Wang et al. (163) found that mouse macrophage-like RAW 264.7 cells stimulated with LPS began to release a nuclear protein, high mobility group box 1 (HMGB1), after the peak release of early inflammatory factors, such as TNF and IL-1. HMGB1 plays a role in inflammatory regulation and stress response; it is an important inflammatory mediator in the late phase of sepsis and a late predictor of mortality in sepsis patients (164, 165). HMGB1 is a non-histone DNA-binding protein, and its function is closely related to its cellular location. Under stimulation conditions such as hypoxia and oxidative stresses, protein post-translationally modified HMGB1 translocates from the nucleus to the cytoplasm or is released into the extracellular space. Acetylation, glycosylation, phosphorylation, ADP-ribosylation, methylation, and redox are the main PTMs of HMGB l. Cys 23, Cys 45, and Cys l06 are key sites for their redox modification, and acetylation and phosphorylation mainly act on HMGB l nuclear localization sequences (NLSs) (164). Under physiological conditions, nuclear HMGBl is a perthiol-type HMGBl in the reduced state. The Cys23, Cys45, and Cysl06 sites connect the thiol side chains (166). ROS generation increases in the perivascular endothelium and tubules of the kidney in an LPS-induced sepsis model. ROS partially oxidize HMGBl to form disulfate-type HMGBl, which confers its cytokine activity and induces inflammatory cells to produce a range of cytokines to promote the inflammatory response (43). In murine macrophage RAW264.7 cells, SIRTl interacts with multiple Lys28-30 on HMGBl to form a complex (154). Lys acetylation after stimulation with LPS and TNF-α leads to conformational changes in HMGBl, separation from SIRTl, and transport to the cytoplasm, followed by the release into the extracellular space, which would subsequently activate downstream inflammatory signaling. In a mouse model of endotoxemia, deacetylation-mediated interaction of SIRT1-HMGB1 improves survival (155).

Histones are highly conserved basic cationic proteins in the cellular chromatin of eukaryotic organisms and are mainly divided into core histones (H2A, H2B, H3, and H4) and linker histones (H1 and H5). The important function of histones is PTMs, including acetylation, methylation, phosphorylation, ubiquitinylation, citrullination, acylation, or glycosylation of ADP. When histones are released into the extracellular space, they are called extracellular histones. The production pathways of extracellular histones include passive necrosis (the membrane is damaged by mechanical trauma or charge- or detergent-related toxicity), NETosis/ETosis (neutrophils/macrophages), necroptosis, pyroptosis, and ferroptosis (167–169). Xu et al. (170) demonstrated that extracellular histones have cytotoxic effects *in vivo* and *in vitro*. In addition, extracellular histones are an endogenous DAMP. By acting on the TLR, they activate various downstream pathways, release many proinflammatory factors (e.g., IL-6, IL-10, TNF-α), and induce platelet aggregation (171, 172). It has been shown that in sepsis patients, circulating histone levels are increased and can cause multiple organ damage (173). This may be caused by a combination of the above mechanisms. There is a close link between the citrullination of histone H3 and sepsis (174). Circulating citrullinated histone H3 (CitH3, a component of NETs) was significantly increased in the CLP-induced mouse sepsis model (156). Inhibitors of protein-arginine deiminase type-4 (PAD4, an enzyme that promotes CitH3 production) modulate this citrullination and reduce CitH3 levels, thereby improving survival in sepsis mice (157). Thus, extracellular histones are involved in the development of sepsis; however, the pathogenic mechanism of their PTMs in sepsis needs further study.

Inflammasomes are protein complexes that promote the maturation and secretion of the cytokines pro-IL-1β and pro-IL-18 by activating caspase-1 (175). There are five inflammasomes, of which the NLRP3 inflammasome plays a key role in sepsis and multiple organ dysfunction as an important component of innate immunity (176). Lee et al. (177) found that NLRP3 deficiency suppressed inflammatory response and improved survival in sepsis mice. Zhong et al. (178) showed that the inhibition of the NLRP3/caspase-1/IL-1β pathway in macrophages attenuated the inflammatory response and microvascular leakage resulting from sepsis. NLRP3 is regulated by a variety of PTMs, including phosphorylation, ubiquitination, alkylation, S-nitrosylation, and ADP-ribosylation (179, 180). Using quantitative proteomics, Stutz et al. (158) found three phosphorylation sites for NLRP3. Among them, they found that the phosphorylation of serine 5 in the pyrin domain inhibits the activation of NLRP3 inflammasome. Phosphatase 2A (PP2A) inhibitors were confirmed using MS to cause increased phosphorylation of serine 5 in LPS-induced macrophages, thereby inhibiting NLRP3 activation. Tang et al. (159) found that RNF125 and Cbl-b (two E3 ubiquitin ligases) sequentially ubiquitinate NLRP3 to inhibit its activation and prevent the development of sepsis in mice. Thus, PTMs of NLRP3, particularly phosphorylation and ubiquitination, regulate its activation and play an important role in sepsis (181).

In sepsis, inflammatory mediators and cytokines induce iNOS production in various cells. NO generated by iNOS combines with the superoxide anion O_2_^–^ to generate peroxynitrite. NO and peroxynitrite can affect the PTM, especially nitrosylation, of cysteine residues in various signaling proteins, such as those involved in excitation-contraction coupling, contraction, energy supply, anti-apoptosis, and anti-oxidative stress (182). S-Nitrosylation refers to the oxidative modification of cysteine by NO to form protein S-nitrosothiols (183). As mentioned above, acetylation of the lysine residues of HMGB1 leads to the release of HMGB1 into the extracellular space (155). SIRT1 activity decreases with increasing S-nitrosylation of SIRT1, resulting in extracellular HMGB1 release (160). Increased levels of iNOS, leading to enhanced production of NO, induce S-nitrosylation of SIRT1 (161). S-Nitroso-N-acetylpenicillamine, as a donor of NO, can increase both S-nitrosylated-SIRT1 levels and the consequent release of HMGB1 (160). NO and NO-mediated PTM can also regulate macrophage immunometabolism (184). Bailey et al. (185) showed that NO modulates the levels of essential TCA cycle-associated metabolites (e.g., citrate and succinate), the inflammatory mediator itaconate, and the complex I subunit in the respiratory chain in inflammatory murine macrophages. NO can also regulate mitochondrial fatty acid metabolism through reversible protein S-nitrosylation (186). During the transformation of LPS-stimulated macrophages from the resting state to the M1 type, the expression of iNOS and NO was increased, and mitochondrial complexes I and IV were modified by nitrosylation, resulting in the destruction of the mitochondrial electron transfer chain and inhibition of OXPHOS (162). Knockdown of iNOS ameliorated LPS-induced mitochondrial respiratory function impairment in M1 macrophages and promoted enhanced OXPHOS. However, treatment of exogenous NO caused mitochondrial dysfunction and promoted macrophage proinflammatory responses (187).

In summary, protein PTMs can affect the development of sepsis by regulating various signaling pathways. At present, proteins with PTMs in sepsis samples can be identified, and the modified amino acid sites can be located using MS combined with bioinformatics analysis.

## Conclusions

Proteomics is a product of the post-genomic era and can be used to study the characteristics of proteins on a large scale. It is mainly used in medicine in the following aspects: 1) to identify markers for the diagnosis or prognosis of a disease, 2) to elucidate the mechanism of the disease and find potential therapeutic targets, and 3) for the classification of diseases. In this review, we have shown that proteomics has made a great contribution to the elucidation of the molecular mechanisms of sepsis; however, it still has some limitations. First, being a molecular technique, there are some inherent challenges. For example, the identification of low-abundance proteins is difficult. The development of protein isolation and identification techniques has provided many effective methods for identifying low-abundance proteins, but they are still not very satisfactory (188, 189). In addition, proteomics cannot be used to detect and identify unknown proteins. Second, biomarkers discovered using proteomics are rarely applied in the clinic, which may be because the number of samples studied is small, and there is high individual heterogeneity. The widespread clinical application of biomarkers requires population-scale validation of their effectiveness. The high cost and time-consuming nature of proteomics is also one of the reasons why most candidate markers are not applied in the clinic. Proteomics is usually employed as the first step in screening biomarkers, but it cannot determine the absolute concentration of proteins present in a sample. Large-scale screening of differentially expressed proteins is performed using high-throughput proteomics, and the sample size then needs to be expanded for the validation of potential biomarkers. Subsequently, western blotting or an enzyme-linked immunoassay is required to determine the expression of candidate biomarkers. Finally, the biomarkers are translated into targets with clinical application value (58). However, there is still a lack of standard techniques and methods for the evaluation and confirmation of the obtained biomarkers to determine their clinical value (58). At present, proteomics research on PTMs mainly focuses on tumors and cardiovascular diseases, and there are few studies on sepsis. In addition, post-translationally modified proteins are low in content in samples and have a wide dynamic range; therefore, enrichment is required to improve the abundance of modified proteins before mass spectrometry (190, 191). In summary, proteomics as a clinical application technology requires much improvement.

Despite the many limitations of proteomics, it is still used to revolutionize our insights into the complex biological processes of sepsis. Moreover, using proteomics in combination with genomics and metabolomics may help comprehensively understand the pathophysiological mechanisms underlying sepsis, determine potential biomarkers, and improve our approach to precision medicine (192).

## Author Contributions

HM designed the review and drafted the manuscript. RD and SC reviewed and revised the article. All authors contributed to the article and approved the submitted version.

## Funding

This study was supported by the grant from LiaoNing Revitalization Talents Program (Grant No. XLYC2007001) and the grant from Changjiang Scholars Program of Ministry of Education of China (TG2019081) and the Natural Science Foundation of Hainan Province (Grant No. 819QN222) and the National Natural Science Foundation of China (Grant No. 81960346, No. 82172174).

## Conflict of Interest

The authors declare that the research was conducted in the absence of any commercial or financial relationships that could be construed as a potential conflict of interest.

## Publisher’s Note

All claims expressed in this article are solely those of the authors and do not necessarily represent those of their affiliated organizations, or those of the publisher, the editors and the reviewers. Any product that may be evaluated in this article, or claim that may be made by its manufacturer, is not guaranteed or endorsed by the publisher.
